# SARS-CoV-2 Seroprevalence in Healthcare Workers before the Vaccination in Poland: Evolution from the First to the Second Pandemic Outbreak

**DOI:** 10.3390/ijerph19042319

**Published:** 2022-02-17

**Authors:** Izabela Korona-Głowniak, Michał Mielnik, Martyna Podgajna, Ewelina Grywalska, Marek Hus, Katarzyna Matuska, Beata Wojtysiak-Duma, Dariusz Duma, Andrzej Glowniak, Anna Malm

**Affiliations:** 1 Department of Pharmaceutical Microbiology, Medical University of Lublin, 20-093 Lublin, Poland; tuskama@wp.pl (K.M.); anna.malm@umlub.pl (A.M.); 2 Department of Hematooncology and Bone Marrow Transplantation, Medical University of Lublin, 20-081 Lublin, Poland; micmiel@gmail.com (M.M.); marek.hus@umlub.pl (M.H.); 3 Department of Experimental Immunology, Medical University of Lublin, 20-093 Lublin, Poland; 50618@student.umlub.pl (M.P.); ewelina.grywalska@umlub.pl (E.G.); 4 Department of Laboratory Diagnostics, Medical University, 20-093 Lublin, Poland; beata.wojtysiak-duma@umlub.pl (B.W.-D.); dariusz.duma@umlub.pl (D.D.); 5 Department of Cardiology, Medical University of Lublin, 20-093 Lublin, Poland; andrzej.glowniak@umlub.pl; 6 Clinical Department of Electrocardiology, SPSK-4 University Hospital, 20-090 Lublin, Poland

**Keywords:** healthcare workers, SARS-CoV-2, IgG/IgA seroprevalence, risk factors

## Abstract

Healthcare workers (HCWs) are on the frontline, struggling with the pandemic caused by severe acute respiratory syndrome coronavirus 2 (SARS-CoV-2). To describe recent or past infections, the serological assays enabled the assessment of the immune response developed in coronavirus disease (COVID-19) in the period when testing was hardly available. In this study, we investigated SARS-CoV-2 seroprevalence in HCWs in a Polish teaching hospital and the Regional Occupational Medicine Center after both the first and the second waves. ELISA-based tests for anti-SARS-CoV-2 IgA and IgG were used to determine immune response to SARS-CoV-2 in volunteer HCWs who worked in those institutions in May 2020 (208 participants aged 47.1 ± 12.5, 88% women) and in December 2020 (179 participants aged 45.2 ± 12.4, 86% woman). Risk factors for seropositivity were also assessed using a questionnaire filled out by all participants. We reported a significant increase in seroprevalence after the second wave (22.9%) compared with the first outbreak (2.4%) (OR 12.1; 95%CI 4.6–31.3; *p* < 0.0001). An association between IgG seroprevalence and severity of infections was noted. Furthermore, we demonstrated that amongst medical personnel, nurses exhibited a proportionally higher SARS-CoV-2 seroprevalence. Moreover, given the high seroprevalence in non-clinical group of HCWs, we suggest that community transmission can play a superior role to workplace exposure.

## 1. Introduction

In December 2019, the biggest pandemic of our century began, triggered by severe acute respiratory syndrome coronavirus 2 (SARS-CoV-2), causing the coronavirus disease 2019 (COVID-19) [1,2]. In Poland, the first imported COVID-19 case was reported on 3 March 2020, and 3 weeks later a nationwide lockdown commenced. Removal of restrictions in May–August for summer appeared to loosen the attitude to public health regulations. Poland was hit by two outbreaks of the pandemic, i.e., from 10 March 2020 to 20 April 2020 and from 4 October 2020 to 27 December 2020, with 1.3 million confirmed SARS-CoV-2 infections and 28.5 thousand deaths caused by the novel coronavirus [3]. Up to 31 December 2021, there were 4.13 million confirmed cases, with 97.6 thousand COVID-19 related deaths [3].

During the rapid spread of the COVID-19 pandemic, 2020 was marked by ongoing research into the disease. Nosocomial transmission may be an important facilitator of infection in epidemics. At the very beginning of the pandemic, The World Health Organization (WHO) recommended molecular reverse transcription PCR (RT-qPCR) testing of respiratory tract samples as a method for the identification and laboratory confirmation of SARS-CoV-2 infection [4]. However, a strategy for fighting the pandemic in Poland was the testing of symptomatic subjects only. Serologic methods based on antibody testing (anti-SARS-CoV-2) can provide a more accurate estimate of epidemic size by detecting diagnosed and undiagnosed cases. Antibody methods rely on detection of IgM, IgG, IgA, or total antibodies by a variety of methods [5]. Then, research also focused on SARS-CoV-2 antibodies detection in the blood of HCWs who are at high-risk of infection [6,7,8,9,10,11]. Initial studies have provided very different estimates of hospital seroprevalence for SARS-CoV-2 in HCWs (ranging from 3.0 to 21.3%) [12,13]. A recent meta-analysis of 11 studies demonstrated that the proportion of HCWs who were SARS-CoV-2 positive among all patients with COVID-19, was 10.1%, but severity and mortality among HCWs were lower than among all patients with COVID-19 [14].

The seroprevalence of SARS-CoV-2 antibodies among HCWs is important information for understanding the spread of COVID-19 among healthcare facilities, and to assess the success of public health interventions. Healthcare is provided in a hospital setting and by family physicians or pediatricians and general practitioners (GPs) who work in a setting away from the hospital.

This study is a serological research performed after the first (May 2020) and second (December 2020) waves of the rise of morbidity in Poland, where most institutions focused on the genetic diagnosis of SARS-CoV-2 infection (vide RT-PCR) to eliminate infected HCWs from the healthcare system. All data collected during abundant research related to COVID-19 contribute to better understanding of disease nature. Here, our objective was to evaluate the prevalence of immunoglobulin G (IgG) and immunoglobulin A (IgA) against SARS-CoV-2 among employees of reference teaching hospital SPSK1 and Regional Occupational Medicine Center (ROMC) to detect what the rates of asymptomatic seroprevalence of SARS-CoV-2 antibodies were during the first and second outbreak in healthcare workers in Southeastern Poland.

## 2. Materials and Methods

### 2.1. Patients and Procedures

This was a prospective study based on voluntary SARS-CoV-2 IgG testing performed among HCWs in Southeastern Poland. Testing was made available for all HCWs (including physicians, nurses, and other workers with direct patient contact, i.e., physical therapists, as well as workers without direct patient contact, i.e., laboratory workers, pharmacists, administrative staff, maintenance staff, etc.) An internet-based recruiting campaign was run, targeting 612 employees of Independent Public Teaching Hospital No. 1 in Lublin and 194 employees of Regional Occupational Medicine Center (ROMC) in Lublin. A total of 208 and 179 healthcare workers responded before the enrollment deadline during the first and the second wave of the pandemic, respectively, and were consequently included into the study. Therefore, the calculated overall response rate was 25.8% during the first, and 22.2% during the second wave. All participants were asymptomatic at the time of serology testing. The history of any flu-like symptoms during the 6-months period preceding testing was obtained. To estimate the severity of the symptoms, a scoring system based on subjects’ self-assessment answers was developed. If the patient did not show any symptoms, a value of 0 was assigned. Each symptom, such as fever, cough, runny nose, fatigue, muscle and joint pain, sore throat, headache, diarrhea and loss of smell or taste, was rated as 2, and period of the flu-like symptoms was rated as the number of days. Additionally, the study participants were asked to estimate the severity of flu-like symptoms in a 10-grade scale. Based on the total sum of values of individual responses, study participants were divided into four groups: “asymptomatic” (0 points), “mild” (1–15 points), “moderate” (16–30 points) or “severe” (31–54 points). 

This study was reviewed and approved by the Medical Ethical Committee of the Medical University of Lublin by resolution No. KE-0254/95/2020.

### 2.2. Laboratory Analysis

The blood samples were collected from individuals and transferred to the Laboratory for Microbiological Diagnostics Medical University of Lublin for analysis of anti-SARS-CoV-2 antibodies presence.

#### Detection of Anti-SARS-CoV-2 Antibodies

ELISA-based tests for anti-SARS-CoV-2 IgA and IgG were from Euroimmun (Lubeck, Germany). These semi-quantitative tests were used per manufacturer’s instructions. Results were calculated as: absorbance value of the sample divided by absorbance value of the calibrators and expressed as extinction ratio. We utilized the manufacturer’s interpretation of the ratio with samples, <0.8 classified as no antibody present, 0.8 ≤ 1.1 indeterminate, and ≥1.1 containing antibodies. These ELISA tests were for antibodies against the S1 subunit/domain of the spike (S) protein of SARS-CoV-2, demonstrate good and excellent specificity for IgA and IgG antibodies.

### 2.3. Statistical Analysis

Continuous variables were presented as median and range. Categorical variables were summarized using percentages and counts. Seroprevalence of SARS-CoV-2 IgG was calculated as a proportion with 95% confidence intervals (CI). The association between variables was tested with the Chi-squared or Fisher’s exact test (for categorical variables) and the Mann–Whitney U test (for continuous variables). Univariable and multivariable logistic regression analysis were run to evaluate factors associated with the seroprevalence of SARS-CoV-2 IgG. For the variables to be included in the multiple logistic model, a stepwise selection was used, starting with the full model, and using a *p*-value of 0.11 for the removal and 0.1 for the addition of variables. A statistical analysis was carried out using the Statistica data analysis software system (TIBCO Software Inc., Tulsa, OK, USA), version 13.

## 3. Results

A total of 387 HCW’s samples from the Independent Public Teaching Hospital No. 1 in Lublin and from Regional Occupational Medicine Center (ROMC) in Lublin were investigated. The studies were carried out in two periods: in the first pandemic outbreak from 5 May to 25 May 2020 (*n* = 208) and in the second outbreak from 4 December to 15 December 2020 (*n* = 179). Subjects’ characteristics at baseline are listed in Table 1. The mean age of recruited volunteers was 47.1 ± 12.5 in May 2020 and 45.2 ± 12.4 in December 2020, of which the majority were woman (88% and 86% in May and December, respectively).

### 3.1. First Observational Period: May 2020

**Figure 1:**
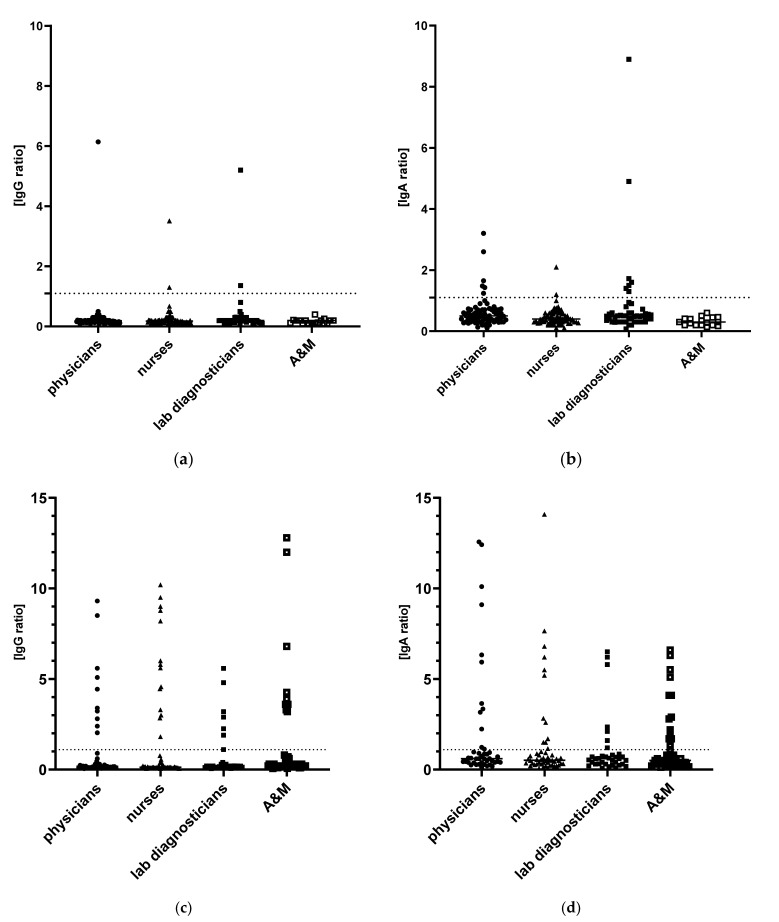
SARS-CoV-2 seroprevalence of IgG (**a**,**c**) and IgA (**b**,**d**) antibodies in recruited healthcare workers stratified by work professional category in first May 2020 (**a**,**b**) and second December 2020 (**c**,**d**) outbreaks.

Positive levels of SARS-CoV-2 IgG and SARS-CoV-2 IgA antibodies were detected in 5 (2.4%) and 15 (7.2%) of 208 subjects, respectively (Table 2). Only two subjects were positive both in specific IgG and IgA antibodies. Two HCWs were positive only in the IgG antibody level and 13 HCWs only in specific IgA antibodies (Figure 1). One physician, two nurses and two lab diagnosticians were SARS-CoV-2 IgG seropositive (*p* = 0.61). SARS-CoV-2 IgA antibodies on diagnostic level were detected in 6 (8.7%) physicians, 2 (2.7%) nurses and 7 (14.3%) lab diagnosticians (*p* = 0.037). None of the tested HCWs had been tested for SARS-CoV-2 infection. There were no statistically significant factors associated with anti-SARS-CoV-2 IgG and IgA antibodies presence in the tested groups. Due to a very low number of seropositive HCWs the study was underpowered to detect statistically significant differences. Immunodeficiency was shown to be a risk factor of IgG seroprevalence (RR 1.25; 95%CI 0.8–1.9; *p* = 0.025); however, it was not confirmed for IgA prevalence (RR 1.1; 95%CI 0.9–1.2; *p* = 0.072) in the group tested during the first observation. Only 2 (7.1%) participants out of 28 who had declared contact with SARS-CoV-2 infected person, were IgG seropositive. The relative risk of IgG seropositivity was higher in ROMC workers than in Teaching hospital personnel (RR 2.6, 95%CI 0.45–15.3, *p* = 0.27) but without statistical significance.

In the first period, the most common symptoms in seropositive IgG cases were myalgia (40.0%), cough (40.0%), asthenia (20.0%), fever (20.0%), sore throat (20.0), and diarrhea (20.0%) (Figure 1). Three out of five IgG positive HCW referred to mild symptoms in the preceding 2 months and two (40%) were asymptomatic.

### 3.2. Second Observational Period: December 2020

Of the 179 HCWs tested in the second period, 41 (22.9%) were seropositive for IgG antibodies to SARS-CoV-2 (Table 2), whereas a positive level of SARS-CoV-2 IgA antibodies was detected in 45 (25.1%) participants (Figure 1). While 69 (38.6%) of HCWs had claimed URT infection in period before sampling, 44 (24.6%) were positively tested against SARS-CoV-2 infection: 24 (13.4%) HCWs in the period at least 1 week before and 20 (11.2%) HCWs were positively tested in a period shorter than 1 week before blood sample collection. A previous positive test was reported by 21 participants, representing 51.2% of the 41 participants with antibodies detected and 90.5% of 21 participants with both antibodies detected and previous URT infection. In the second observation, IgG concentration was positively correlated with the infection index reported by 69 participants (Spearman R = 0.47; *p* < 0.0001); then IgA concentration was positively correlated with both infection index (Spearman R = 0.44; *p* < 0.0001) and age (Spearman R = 0.17; *p* = 0.026).

As the study population worked in the hospital or ROMC, 22.9% (41 HCWs) had contact with COVID-19 patients. Nevertheless, the percentage of IgG and IgA positive cases did not differ significantly between the subjects with a history of contact with COVID-19 patients in comparison with non-contacts (27.8% vs. 15.5% for IgG, *p*-value: 0.069; 30.6% vs. 16.9% for IgA, *p*-value: 0.052). Moreover, there were no significant differences in IgG and IgA positive cases between hospital and ROMC workers (22.7% vs. 23.7% for IgG, *p* = 1.0 and 24.1% vs. 29.0% for IgA, *p* = 0.53). A specific hospital job was not associated with an increased proportion of IgG and IgA positivity: nurses presented higher IgG percentage (27.5%), whereas other professional categories than medical doctors and nurses had higher IgA prevalence (30.0%) (Table 2).

**Figure 2:**
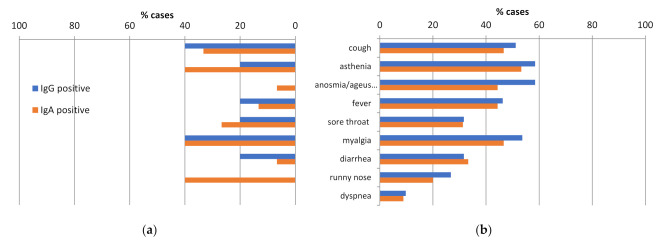
Symptom description of seropositive HCWs. (**a**) May 2020; (**b**) December 2020.

In the second test period, the most common symptoms in seropositive IgG cases were myalgia (53.7%), cough (51.2%), asthenia (58.5%), and fever (46.3%), similarly as the first period. Moreover anosmia/ageusia (58.5%) was very common (Figure 2). Contrary to the first period, 20 out of 41 (48.8%) IgG positive HCWs referred to severe symptoms in the 6 months before testing and 8 (19.5%) were asymptomatically infected. We observed a difference in seropositivity among prior symptomatic and asymptomatic HCWs (47.8% vs. 7.3%).

### 3.3. Risk Factors and Seropositivity—Multivariate Analysis

We evaluated factors associated with SARS-CoV-2 seropositivity by logistic regression analysis. Variables included in the model of the logistic regression analysis were presented in Table 3. Regarding professional categories, HCWs with an increased probability of SARS-CoV-2 IgG positive test were nurses (OR 5.0, 95%CI 0.85–29.3), and lab diagnosticians (OR 2.87, 95%CI 0.5–16.1). Regarding working areas, the ROMC area (OR 1.53, 95%CI 0.36–6.55) was associated with increased risk of seropositivity, albeit insignificantly. Male participants were significantly more frequently at risk of IgG seropositivity (OR 7.5, 95%CI 1.4–41.0) together with higher infection index (OR 1.18, 95%CI 1.08–1.3) and gastrointestinal disease (OR 42.5, 95%CI 1.2–1455.7). In a logistic regression model, the latter two were confirmed as factors significantly associated with SARS-CoV-2 infection in the past and IgG antibody presence.

Moreover, the univariate analysis indicated endocrinological disease as positively associated with IgG positive detection (OR 2.44, 95%CI 1.1–5.45), yet it was not confirmed in the multivariate analysis.

## 4. Discussion

This is one of a very few reports regarding the SARS-CoV-2 seroprevalence in Poland, which is important considering the period when testing was hardly available and the different, country-specific approach to the pandemic control. We reported the results of a SARS-CoV-2 seroprevalence study conducted among HCWs from two workplaces in two following pandemic outbreaks in Poland. We found an overall seroprevalence of SARS-CoV-2 antibodies of 2.4% in the first pandemic wave and significant increase in seropositive HCWs in the second wave to 22.9% (OR 12.1; 95%CI 4.6–31.3; *p* < 0.0001). There were significant differences in seroprevalence associated with the severity of previous infection and comorbidities with gastrointestinal tract, but no differences associated with professional cadre.

According to the World Health Organization (WHO), one out of every seven COVID-19 patients (14%) is an HCW [15]. In this study, we estimated the seroprevalence of HCWs in the first wave as lower than those in most of studies from West European countries published to date, which were conducted in highly impacted countries during the first pandemic wave [16,17,18]. A pooled seroprevalence from the United States, ten European, and three East Asian countries was estimated as 8.6% (95%CI = 7.2–9.9%) [19]. Seroprevalence was higher in studies conducted in North America (12.7%) compared with those conducted in Europe (8.5%), Africa (8.2), and Asia (4%) [20].

The national healthcare institutions in Poland were impacted by the pandemic less than in the majority of Western European countries and the spread of COVID-19 was classified early by the World Health Organization as community transmission. In Poland, at the very beginning, extensive solutions were implemented to reduce the spread of infection. In Poland, 6 days after the first laboratory-confirmed COVID-19 case, all mass events were forbidden, at 8 days after, all schools and universities were closed. After 11 days, major travel restrictions and the 14 days self-quarantine obligation for travelers were introduced [21]. By August 2020, in Poland about 60,000 COVID-19 cases with 2000 related deaths had been detected in comparison to Spain (above 386,000 cases), France (above 272,000 cases), or Italy (above 257,000 cases), where the pandemic situation required the rapid reorganization of national healthcare systems [22].

After the first pandemic wave, retrospective analysis of voluntary SARS-CoV-2 IgG testing in Poland was provided free of charge to HCWs of the Children’s Memorial Health Institute in Warsaw from July to August 2020. A large group of 1879 HCWs (82.3% of eligible participants) volunteered to undergo testing. SARS-CoV-2 IgG seroprevalence of 0.85% was detected [23]. Other results from Poland showed the IgG seropositivity of asymptomatic HCWs presented by 90 participants from Hospital No. 4 in Bytom and 84 HCWs in the University Hospital in Opole and 25 in a Miasteczko Śląskie local surgery. The local diversity observed varied between 1.2% and 10% (Opole vs. Bytom, *p* < 0.05; all without any symptoms). IgA seropositivity in HCWs was 8.8% in Opole and 7.14% in Bytom [24]. In the study carried out in Poznań (Poland) after the first wave of COVID-19 (July-September 2020), based on the ELISA results, it was found that 1.67% (95%CI 1.13–2.45) had antibodies against SARS-CoV-2 [25]. However, there were regions in Poland highly affected by COVID-19 and in a cross-sectional study located in three large towns of Silesian Voivodeship (Poland), the authors studied 5479 subjects in which 1253 (22.9%) had a positive anti-SARS-CoV-2 IgG test [26].

This pandemic depiction in Poland is quite distinct from European countries which were highly impacted by pandemic. Seroprevalence among European studies in HCWs ranged from 0.4% to 45.3%, namely UK (6.0–45.3%); Spain (9.3–31.3%); Sweden (19.1%); Belgium (6.4–14.6%); Italy (0.4–5.1%), and France (5.3%) [20]. However, there were countries such as Greece (1.0%) and Germany (1.4–2.6%) with a low burden of COVID-19 during the first wave where seroprevalence among HCWs is analogous to our results [27,28].

Such low prevalence in the first wave let us presume that this population was vulnerable to the next outbreak of the COVID-19 pandemic, that was indeed observed in the second wave. In Poland, in the period October–November 2020, the prevalence of SARS-CoV-2 infection was larger than earlier estimates obtained in other European countries, probably reflecting the measurements obtained during the second wave of the epidemic. The prevalence of IgG seropositivity was 11.4% (95%CI: 9.5–13.2%). Moreover, the second wave of the COVID-19 epidemic in Poland started in September 2020 and the measurements showed that the frequency of positive IgG tests increased from 5.6% in October to 15.0% in November [29].

In our study, after the second outbreak, seroprevalence among HCWs was much higher than in the first wave and reached 23% in tested HCWs. The probable explanation can be the rapid spread of the virus during summer among the general population, which led Polish hospitals to dedicate entire wards to confirmed COVID-19 cases, leading to greater high-risk contacts for HCWs. Surprisingly, HCWs with no direct contact with patients such as non-clinical workers (20.0%) had higher seroprevalence than the general population in Poland [29]. These data suggest a role of nosocomial transmission in non-clinical workers [30]. In these HCW cases, other HCWs are the likely source of infection rather than the patients. Moreover, in our study, there were no differences between the seroprevalence of hospital workers (22.7%) and ROMC workers (23.7%). The absence of differences in seroprevalence by a professional category suggests that community transmission can be play a bigger role than workplace exposure. In studies of HCWs conducted in the United Kingdom, the incidence of infection mirrored that observed in the community [31,32]. Seroprevalence pattern findings from other studies in healthcare workers indicated interlocking different exposure risks from the work environment, home environment, and barely measured community-based factors [11]. This suggests clearly that efforts to suppress community transmission are probable to reduce infections among HCWs.

Although the high percentage of asymptomatic cases among HCWs with evidence of SARS-CoV-2 infection (56.3%) was observed in previous report [23], in this study, both in the first and second waves about 20% of such cases were detected, which is in the line with other reports [31,33].

Lack of wide-reaching testing in the first wave of the pandemic did not allow to assess the real scale of SARS-CoV-2 infections in HCWs. Thus, seroprevalence studies conducted in high-risk subgroups are useful to assess the level of exposure and identify risk factors of SARS-CoV-2 infection in this population. 

This study depicted the low burden of infection in our region, and thus the study is underpowered for pointing out risk factors in the first pandemic outbreak, but we performed analyses for the second observational period. Several studies analyzed risk factors for SARS-CoV-2 seropositivity in HCWs with different results [11,20,34]. In the meta-analysis by Hossain et al. [19] the overall pooled odds ratio of 25 studies for the association between gender and IgG antibody status was 1.18 (OR = 1.18, 95%CI = 1.06–1.31) indicating the odds of catching an infection in male HCWs was higher by 18% than female HCWs. In our study, the risk was 7.5 times higher for males than females. The systematic review and meta-analysis were conducted to estimate the sex difference in acquiring COVID-19 [35]. This indicated that the behavioral factors and social roles which increase the risk of acquiring COVID-19 tend to be more common among men. In most research, the prevalence of IgG antibodies per age-group was absent [19]. This was also convergent to our study. Even though, in our study, participants older than 50 years were more frequently SARS-CoV-2 IgG seropositive, statistical significance was not observed (OR 1.8; 95% 0.9–3.6; *p* = 0.11).

We observed more robust immunity responses in the HCWs with increasing severity of infection. Similar to this study, most studies have described higher levels of IgG antibodies among those with more severe disease, and some have suggested that a high level of IgA response, in particular, may have a pathological role in SARS-CoV-2 infection [36,37,38].

The majority of HCWs in this study reported mild and moderate symptoms which are difficult to distinguish from other respiratory infections. The identification of symptoms predictive of SARS-CoV-2 infection, single or in combination, and their severity is essential for the screening guidance and the recommendations of self-isolation for epidemic reason. In the large population-based study from the United States, fever, cough, and dyspnea were reported as hallmark symptoms of COVID-19 infection [39,40]. In this study, fever, cough, anosmia, and ageusia were the most common symptoms among the seropositive HCWs. The results from the present study are in line with the recent large multinational population-based cohort study investigating potential symptoms of COVID-19, reporting a strong association between anosmia, ageusia and COVID-19 [41,42]. Furthermore, a strong association between the symptom combinations anosmia and ageusia (OR 56.8, 95%CI 11.5–282.1, *p* < 0.0001), fever (OR 4.4, 95%CI 1.2–15.6, *p* = 0.023), cough (OR 5.2, 95%CI 1.6–17.4, *p* = 0.008), and seroprevalence was found, suggesting that these symptoms should be included in routine screening guidance.

Despite recent improvements in COVID-19 management and vaccine development, still no clear results and future perspectives can be drawn. This study shows how seroprevalence in exposed HCWs evolved during the COVID-19 pandemic from the first wave to the second wave, in a context of increasing viral circulation leading to the first encounter of SARS-CoV-2. Future seroprevalence studies in HCWs will bring further insights on the pandemic evolution noting vaccine-related seroprevalence. To date, 35% of citizens in Poland are unvaccinated, meaning there is still a large number of sensitive individuals who present a high risk of acquiring infection.

This study has several limitations. Firstly, we collected data by nonrandom selection of only a small proportion of the HCWs in the region. This could lead to an overestimation of the seroprevalence if the HCWs sampled had an overrepresentation of individuals who had experienced symptoms in the past, since it is a two-center design with voluntary participation and subject characteristics collected via a self-reported online survey. Moreover, data regarding symptoms within the previous 12 weeks were collected, resulting in the possibility of self-report bias. Taking into account the big variation in regional SARS-CoV-2 seroprevalence in Poland, the data from the general population seroprevalence in our region would be usable for comparison.

## 5. Conclusions

We documented SARS-CoV-2 seroprevalence in HCWs in two medical units: a university teaching hospital and the Regional Occupational Medicine Center after both the first and second waves of infections during the COVID-19 pandemic in 2020. Our data demonstrates a pointed increase in HCWs seroprevalence in these institutions within test periods and describes the evolution in seroprevalence as well as an association with severity of symptoms and higher risk of seropositive anti-SARS-CoV-2 IgG in people with gastrointestinal tract diseases in the second wave. The lack of significant differences in seroprevalence by professional category suggests that community transmission can play a superior role to workplace exposure.

## Figures and Tables

**Table 1:** Demographics. General description of the participants in the study.

Characteristics	May 2020 Total No = 208	December 2020 Total No = 179
Women	183 (88.0%)	154 (86.0%)
Age (mean ± SD) years	47.1 ± 12.5 (49.0; 24–74)	45.2 ± 12.4 (46.0; 21–69)
Age groups		
18–33 years	48 (23.1%)	42 (23.5%)
34–49 years	61 (29.3%)	61 (34.1%)
50–64 years	83 (39.9%)	68 (38.0%)
65+	16 (7.7%)	8 (4.5%)
Clinical conditions (comorbidities)	86 (41.4%)	82 (45.8%)
Respiratory tract disease	8 (3.8%)	7 (3.9%)
Endocrinological disease	39 (18.8%)	35 (19.6%)
Cardiovascular disease	36 (17.3%)	35 (19.6%)
Diabetes mellitus	4 (1.9%)	4 (2.2%)
Immunodeficiency	1 (0.5%)	3 (1.7%)
Allergy	34 (16.3%)	21 (11.7%)
Gastrointestinal tract disease	6 (2.9%)	2 (1.1%)
Cancer	2 (1.0%)	2 (1.1%)
Occupational SARS-CoV-2 exposure	28 (13.5%)	108 (60.3%)
Previous positive PCR test	0 (0%)	24 (13.4%)
URT Infection in previous 3 months	81 (38.9%)	69 (38.5%)
Infection index		
Mild (≤15 points)	23 (11.1%)	15 (8.4%)
Moderate (16–30 points)	48 (23.1%)	33 (18.4%)
Severe (≥31 points)	10 (4.8%)	21 (11.7%)
Professional category		
Administrative and management	14 (6.7%)	50 (27.9%)
Laboratory diagnostician	51 (24.5%)	35 (19.6%)
Nurse	71 (34.1%)	51 (28.5%)
Physician	69 (33.2%)	43 (24.0%)
Workplace		
Regional Occupational Medicine Center	42 (20.2%)	38 (21.2%)
Teaching Hospital	166 (79.8%)	141 (78.8%)

URT: upper respiratory tract.

**Table 2:** IgG and IgA seroprevalence rates in participants in two observational studies.

Characteristics	May 2020 Total No = 208		December 2020 Total No = 179	
	Positive IgG (*n* = 5)	Positive IgA (*n* = 15)	Positive IgG (*n* = 41)	Positive IgA (*n* = 45)
Women	5 (100%)	13 (86.7%)	34 (82.9%)	37 (82.2%)
Men	0 (0%)	2 (13.3%)	7 (17.1%)	8 (17.8%)
Age groups				
18–33 years	1 (20.0%)	3 (20.0%)	9 (21.9%)	9 (20.0%)
34–49 years	2 (40.0%)	5 (33.3%)	10 (24.4%)	13 (28.9%)
50–64 years	2 (40.0%)	6 (40.0%)	18 (43.9%)	19 (42.2%)
65+	0 (0%)	1 (6.7%)	4 (9.8%)	4 (8.9%)
Clinical conditions (comorbidities)	3 (60.0%)	6 (40.0%)	18 (43.9%)	21 (46.7%)
Respiratory tract disease	0 (0%)	0 (0%)	0 (0%)	0 (0%)
Endocrinological disease	1 (20.0%)	2 (13.3%)	13 (31.7%)	14 (31.1%)
Cardiovascular disease	1 (20.0%)	3 (20.0%)	5 (12.2%)	8 (17.8%)
Diabetes mellitus	0 (0%)	0 (0%)	1 (2.4%)	1 (2.2%)
Immunodeficiency	1 (20.0%)	1 (6.7%)	0 (0%)	0 (0%)
Allergy	0 (0%)	2 (13.3%)	3 (7.3%)	3 (6.7%)
Gastrointestinal tract disease	0 (0%)	1 (6.7%)	1 (2.4%)	1 (2.2%)
Cancer	0 (0%)	0 (0%)	0 (0%)	0 (0%)
Occupational SARS-CoV-2 exposure	2 (40.0%)	3 (20.0%)	30 (73.2%)	33 (73.3%)
Previous positive PCR test	0 (0%)	0 (0%)	21 (51.2%)	18 (40.0%)
URT Infection in previous 3 months	4 (80.0%)	10 (66.7%)	33 (80.5%)	35 (77.8%)
Infection index				
Mild (≤15 points)	3 (60.0%)	4 (26.7%)	3 (7.3%)	4 (8.9%)
Moderate (16–30 points)	1 (20.0%)	3 (20.0%)	10 (24.4%)	14 (31.1%)
Severe (≥31 points)	0 (0%)	3 (20.0%)	20 (48.8%)	17 (37.8%)
Professional category				
Administrative and management	0 (0%)	0 (0%)	10 (24.4%)	15 (33.3%)
Laboratory diagnosticians	2 (40.0%)	7 (46.7%)	7 (17.1%)	6 (13.3%)
Nurse	2 (40.0%)	2 (13.3%)	14 (34.1%)	12 (26.7%)
Physician	1 (20.0%)	6 (40.0%)	10 (24.4%)	12 (26.7%)
Workplace				
ROMC	2 (40.0%)	4 (26.7%)	9 (21.9%)	11 (24.4%)
Teaching Hospital	3 (60.0%)	11 (73.3%)	32 (78.0%)	34 (75.6%)

ROMC: Regional Occupational Medicine Center; URT: upper respiratory tract.

**Table 3:** Univariate and multivariate analysis of factors associated with SARS-CoV-2 IgG positivity.

Characteristics	Univariate		Multivariate (All Effects)		Multivariate (Model)	
	OR (95%CI)	*p*	OR (95%CI)	*p*		
Men	1.37 (0.53–3.56)	0.51	7.5 (1.4–41.0)	0.021		
Age	1.02 (0.99–1.05)	0.17	1.02 (0.98–1.08)	0.29		
Clinical conditions						
Respiratory tract disease	0.0 (0.0)	1.0	0.0 (0.0)	1.0		
Endocrinological disease	2.44 (1.1–5.45)	0.028	1.5 (0.37–5.8)	0.58		
Cardiovascular disease	0.50 (0.18–1.39)	0.18	0.26 (0.04–1.59)	0.15		
Diabetes mellitus	1.13 (0.11–11.1)	0.92	1.36 (0.04–44.2)	0.86		
Immunodeficiency	2.5 (0.26–24.6)	0.43	0.0 (0.0)	1.0		
Allergy	0.0 (0.0)	1.0	0.27 (0.03–2.4)	0.24		
Gastrointestinal tract disease	3.42 (0.2–55.9)	0.39	42.5 (1.2–1455.7)	0.038	19.2 (0.86–426.0)	0.043
Occupational SARS-CoV-2 exposure	2.1 (0.97–4.52)	0.059	1.54 (0.48–4.9)	0.47		
URTI in previous 3 months	11.7 (4.9–27.6)	<0.0001	0.35 (0.03–3.7)	0.38		
Infection index	1.1 (1.08–1.2)	<0.0001	1.18 (1.08–1.3)	<0.0001	1.1 (1.06–1.15)	<0.0001
Professional category						
Administrative and management	0.8 (0.31–2.22)	0.70	1.72 (0.35–8.4)	0.5		
Laboratory diagnosticians	0.8 (0.28–2.45)	0.84	2.87 (0.5–16.1)	0.23		
Nurse	1.2 (0.49–3.2)	0.46	5.0 (0.85–29.3)	0.075		
Physician	referent		referent			
Workplace						
ROMC	1.06 (0.45–2.46)	0.9	1.53 (0.36–6.55)	0.57		
Teaching Hospital	referent		referent			

ROMC: Regional Occupational Medicine Center; URTI: upper respiratory tract infections; OR: odds ratio; 95%CI: 95% confidence interval.

## Data Availability

Due to privacy and ethical concerns, the data that support the findings of this study are available on request from the First Author, [I.K.-G.].
